# Bayesian Sigmoid-Type Time Series Forecasting with Missing Data for Greenhouse Crops

**DOI:** 10.3390/s20113246

**Published:** 2020-06-07

**Authors:** Alexander Kocian, Giulia Carmassi, Fatjon Cela, Luca Incrocci, Paolo Milazzo, Stefano Chessa

**Affiliations:** 1 Department of Computer Science, University of Pisa, 56127 Pisa, Italy; paolo.milazzo@unipi.it (P.M.); stefano.chessa@di.unipi.it (S.C.); 2 Department of Agriculture, Food and Environment, University of Pisa, 56124 Pisa, Italy; giulia.carmassi@unipi.it (G.C.); fatjon.cela@agr.unipi.it (F.C.); Luca.incrocci@unipi.it (L.I.)

**Keywords:** prediction, sigmoid, time-series, dynamic Bayesian network, missing data, evapotranspiration, leaf area index, MicroTom, IoT

## Abstract

This paper follows an integrated approach of Internet of Things based sensing and machine learning for crop growth prediction in agriculture. A Dynamic Bayesian Network (DBN) relates crop growth associated measurement data to environmental control data via hidden states. The measurement data, having (non-linear) sigmoid-type dynamics, are instances of the two classes observed and missing, respectively. Considering that the time series of the logistic sigmoid function is the solution to a reciprocal linear dynamic model, the exact expectation-maximization algorithm can be applied to infer the hidden states and to learn the parameters of the model. At iterative convergence, the parameter estimates are then used to derive a predictor of the measurement data several days ahead. To evaluate the performance of the proposed DBN, we followed three cultivation cycles of micro-tomatoes (MicroTom) in a mini-greenhouse. The environmental parameters were temperature, converted into Growing Degree Days (GDD), and the solar irradiance, both at a daily granularity. The measurement data were Leaf Area Index (LAI) and Evapotranspiration (ET). Although measurement data were only available scarcely, it turned out that high quality measurement data predictions were possible up to three weeks ahead.

## 1. Introduction

The need to supply food to a growing population leads agriculture to deal with new and significant challenges. High-input and resource-intensive farming systems, which have caused massive deforestation, water scarcities, soil depletion, and high levels of greenhouse gas emissions, cannot deliver sustainable food and agricultural production. Instead, agriculture will increasingly need innovative systems that protect and enhance the natural resource base, while increasing productivity [1]. Precision agriculture has a significant potential to reduce agricultural inputs, enhance agricultural sustainability, and increase production, as it allows greater accuracy in targeting the correct amount of inputs, at the correct time, and in the correct location compared to conventional agricultural methods [2]. Within the precision agriculture approach are currently also included Internet of Things (IoT) technologies [3]. In a recent review [4], the authors analyzed the existing literature on the concept of IoT applications in precision agriculture, and they concluded that IoT may contribute significantly to modern agriculture, by providing automated and even remote control of farms, thus enabling a more effective management of agricultural activities, in this innovation supported by new age farmers that have already moved away from traditional farming to engage with technological farming. However, with the introduction of IoT comes the problem of interpreting the large amount of data that are produced with this technology, which passes through to the need to develop new models capable of processing capillary and high resolution data, a requirement that can be met by the massive introduction of data-driven Artificial Intelligence (AI) techniques [5,6,7]. IoT technologies are also suitable for use in the greenhouse production systems where the environment is controlled, often equipped with sensors to monitor environmental and crop parameters, and the data can be acquired and processed [8]. Recent advancements in IoT, AI, and Information and Communication Technologies (ICT) in general have the potential to address some of the environmental, economic, and technical challenges, as well as opportunities in this sector [9]. Protected cultivation has rapidly expanded in many regions all over the world [10]. This cultivation system is constantly evolving; progress is continuous; and the environmental concerns are increasingly important in the greenhouse production system. New approaches and technologies are oriented toward reducing any environmental impact and an optimal management of the input. Real-time estimation of the input and the combining of a monitoring approach and modeling are growing in importance [10]. In a recent review on the impact of protected vegetable cultivation [11], the importance of good agricultural practices, especially with respect to irrigation or fertilization, was reported for the reduction of greenhouse gas emissions.

The applications of AI technologies in agriculture are many and cover several different aspects, addressing crop, water, and soil management. Concerning crop management, the applications span from yield prediction to the recognition and classification of diseases, weed detection, crop quality, species recognition, etc. [5]. A commonly used technique in agriculture is Support Vector Regression (SVR) [12,13]. Equipped with Radial Basis Functions (RBFs) as the kernel, the SVR in [14] was capable of predicting non-linear rice yield after training with historical data covering a period of 20 years. Furthermore, back-propagation and RBF neural networks have been successfully applied to (non-linear) price forecasting in agriculture after a training period of 365 days [15]. Note that the size of the training set needs to be at least one magnitude larger than that of the feature space. Among several AI methods, the Bayesian approach is particularly appealing due to its ability to make accurate predictions even with limited datasets. Here, a statistical model for the crop growth turns a first guess of the state-variable distributions, i.e., the prior distribution, along with measurements into a posterior distribution. The parameters of the model are assumed to be known. As a matter of a fact, Bayesian models have been successfully used to predict fruit yield [16] and disease development [17,18]. As concerns this work, we are interested in AI models suitable for predicting the growth of crops that may allow farmers to better plan their control actions, optimize the input, and quantitatively evaluate (through simulations) the effects of alternative interventions when the model parameters are unknown. A significant work on how historical growth, soil characteristics, and environmental parameters can be used to predict the time series of crop growth-related indicators such as Leaf Area Index (LAI) and evapotranspiration rate was given in [19]. However, the self-learning model only supported crops with exponential growth dynamics. Furthermore, the measurement data must be complete.

Nevertheless, some of the measurement data may be missing due to sensor failure, the battery’s low charge, or the measurement process (for example, destructive samples are only available in a limited quantity). Missing data randomness can be divided into three classes: (i) Missing At Random (MAR): The probability that an observation is missing only depends on the observed data, but is independent of the missing data. When the model parameters are independent of the parameters of the missing data, the missing data mechanism is ignorable [20]. (ii) Missing Completely At Random (MCAR), a special case of MAR, where the probability that an observation is missing is independent of the observed and missing data. (iii) Not missing a random is data that are neither MAR nor MCAR.

Most data analysis techniques in the literature, including SVR and neural networks, have no native mechanism that handles missing data and, hence, exclude observations with missing variable values, introducing a bias. Another strategy is simple imputation, in which each missing value is substituted [21]. For example, each missing value can be imputed by interpolation techniques (for example, linear, quadratic, cubic, and nearest neighbor interpolation) [22]. Following this approach, missing values are treated as they were known in the analysis. Consequently, the bias is reduced. Multiple imputation, in contrast, samples multiple values from the probability distribution of observed sensor data and takes the statistical mean, variance, and confidence interval to fill the gap [20]. A standard approach for multiple imputation is the Data Augmentation (DA) algorithm, which is a Markov chain Monte Carlo technique [23]. In Bayesian inference, the missing data can be resolved by iterating between the imputation step and posterior step [21]. The most efficient method, we follow subsequently, is to approach the maximum likelihood estimate in the presence of missing data using the expectation-maximization algorithm [24]. In contrast to the stochastic approximation in [25], our solution is exact.

In this study, we develop a DBN for time series forecasting with daily granularity of crop growth in the IoT based mini-greenhouse COLTIVazione Automatizzata Miniaturizzata Innovativa (COLTIV@MI) [26]. Our DBN learns the model parameters ad-hoc without the need for time consuming training cultivation cycles. Our DBN relates the non-linear crop growth associated measurement data LAI and ET to the environment associated control data GDD and solar irradiance via hidden states. Many plants have a logistic growth evolution. To resemble such growth dynamics, the DBN will be based on a highly complex non-linear model. We will show that the reciprocal function, though, follows a linear dynamic model with low computational complexity. Often, measurement data are only available scarcely at an irregular time spacing. For example, the LAI has been obtained by destructive measurement once every three weeks. To learn the parameters of the model in Gaussian noise when measurement data are MAR, we derive a novel tracking algorithm based on the exact Expectation-Maximization (EM) framework. The self-trained model is then used to derive a measurement predictor many days ahead. As an example, we consider the micro-tomatoes MicroTom [27] through the proposed approach, which can be applied to any other crop type with sigmoid-type growth dynamics. We will see that LAI and ET can be predicted up to 21 days ahead with high accuracy even if almost all of the measurement data are missing.

The paper is organized as follows. Section 2 develops the DBN, the underlying system model, and the EM algorithm to learn the parameters of the model. The plant growth parameter estimates at iterative convergence are then used to derive a multiple-step ahead predictor. The experimental settings will be discussed. The experimental results are discussed in Section 3 followed by conclusions and limitations in Section 4.

## 2. Materials and Methods

### 2.1. The Standard Models and Beyond

The LAI is a dimensionless measure for the total area of leaves per unit projected ground area and directly related to the amount of light that can be intercepted by plants. The LAI is a key parameter to predict photosynthetic biomass production. For tomatoes, it was shown in [28] that LAI from planting to the time of harvest can be modeled as a function of thermal time, expressed in growing degree days (GDD) (°C). The resulting function:

(1) $${\mathrm{LAI}}_{t}=\alpha +\frac{\beta -\alpha }{1+\exp\{\left(\zeta -\sum_{t^{\prime }\leq t}{\mathrm{GDD}}_{t^{\prime }}\right)/\delta \}}.$$

has the form of a Boltzmann sigmoid where α, β, ζ, and δ are constants to be obtained by regression analysis. The index *t* in (1) determines the $t^{\mathrm{th}}$ Growing Day after Transplantation (GDT). For the calculation of GDD from average daily temperature, a value of 10 °C was selected as a base temperature according to [29].

The Evapotranspiration (ET) is a combination of the water transpired by plants during the growth or retained in the plant tissue plus the moisture evaporated from the soil surface and vegetation. Several ET models have been developed during the last few years, all based on the Penman–Monteith approach [30], which is a worldwide accepted modeling approach to determine evapotranspiration. The current use of the Penman–Monteith model is based on the calculation of ET for outdoor climates, while the Stanghellini ET model [31] was implemented in high technology controlled environment greenhouses. Both equations require several parameters related to the weather, as well as the crop stomatal and aerodynamic resistances, which are not always available. Alternative relatively simple empirical models have been developed for irrigation scheduling purposes, while the other knowledge based mechanistic models have been developed for climate control purposes [32]. These models take into account greenhouse climate variables such as radiation and vapor pressure deficit and crop measurements such as the Leaf Area Index (LAI) or stomatal resistance [28,33,34,35,36,37,38,39]. In some cases, a multiple linear regression of ET against vapor pressure deficit, as well as outside or inside solar radiation has been proposed for irrigation management in greenhouse crops [40]. For tomatoes, it was shown in [28] that the Baille model in [33] simplifies to:

(2) $${\mathrm{ET}}_{t}=\epsilon \left(1-\exp\{-k\;{\mathrm{LAI}}_{t}\right\})\frac{R_{t}}{\lambda }+\gamma .$$

The ET is expressed in mm d${}^{-1}$; $R_{t}$ is the daily value of solar irradiance (MJ m${}^{-2}$ d${}^{-1}$); λ is the latent heat of water vaporization (2.45 MJ kg${}^{-2}$); *k* is the light extinction coefficient of the canopy (measured as 0.69); and ϵ and γ are the regression parameters. Inspecting (2), it can be seen that the ET is a modulated sigmoid function with a negative exponential envelope. The models (1) and (2) act as the benchmark for our dynamic Bayesian growth model.

Difference equations can be used to predict the growth status one discrete time step ahead. Specifically, the time series of the LAI in (1) resembles the scaled and time-shifted logistic function $z_{t}={\left[\kappa \left(1+\exp\left\{-\mu \left(t-t_{0}\right)\right\}\right)\right]}^{-1}$ with the system parameters κ, μ, and $t_{0}$ denoting the steady state, decay, and the point of inflection, respectively. The logistic function is the solution to a non-linear difference equation, which is cumbersome to implement. Its reciprocal dynamics,

(3) $$x_{t}≜z_{t}^{-1}=\kappa \left(1+\exp\left\{-\mu \left(t-t_{0}\right)\right\}\right),$$

however, has an exponential shape that can easily be generated by the first-order linear ordinary difference equation:

(4) $$\frac{x_{t+1}-x_{t}}{\Delta }=-\mu \;x_{t}+\kappa \mu$$

at low computational complexity. Here, Δ denotes the time granularity. Note that the first term on the right-hand side of (4) depends on the latent variable, while the second does not. On the other hand, the ET time series in (2) has the form:

(5) $$x_{t}=\kappa^{\prime }\left(1-\exp\left\{-\mu^{\prime }t\right\}\right).$$

Note that despite the different sign in front of the exponential, the expressions in (3) and (5) are the solution to the same difference equation in (4), but with distinct initial conditions.

### 2.2. Dynamic Bayesian Network

We now derive a Dynamic Bayesian Network (DBN) for stochastic crop growth with the sigmoid-type activation function when observation data are sparse. When the underlying process is Markovian, the DBN replicates a particular template over discrete steps in time. The template is a directed acyclic graph, representing the state transition distribution from one state to the next state and the emission distribution within the same state. The edges of the DBN reflect a conditional dependency, while the nodes correspond to one of the three kinds of variables. The arrows indicate the conditional dependencies. The expression in (4) motivates us to model the reciprocal dynamics of crop growth as DBN, having a length of *T* growing days. We subsequently define the states, derive the template, and draw the DBN.

For the states, the transition probability distribution from hidden growth state $\left\{x_{t}:x_{t}\in R^{K},t\in \left[1,T\right]\right\}$ to state $x_{t+1}$,

(6) $$p\left(x_{t+1}|x_{t}\right)=N\left(x_{t+1};\left(I-A\;U_{t}\right)x_{t}+B\;u_{t},\Sigma_{n}\right),$$

follows a first-order Markov process with additional Gaussian noise. Here, N(·;m,Σ) denotes a normal distribution with mean m and covariance Σ. Moreover, $A\in R^{K\times K}$ and $B\in R^{K\times K}$ denote the state matrix and the control matrix, respectively; I is the *K*-dimensional identity matrix, and the diagonal matrix $U_{t}≜\mathrm{diag}\left\{u_{t}\right\}$. The control vector $u_{t}$, $u_{t}\in R^{K}$ is a deterministic function of the environmental parameters independent of the crop.

The template of the DBN is comprised of the above state distribution and subsequent emission distribution:

(7) $$p\left(y_{t}^{\left(\mathrm{obs}\right)}|x_{t}\right)=N\left(y_{t}^{\left(\mathrm{obs}\right)};{Cx}_{t},\Sigma_{w}\right)$$

instead of having perfect knowledge of the state variable. Here, the measurement matrix is denoted by $C\in R^{1\times K}$. Moreover, $y_{t}^{\left(\mathrm{obs}\right)}\in R$ denotes the observed data. The remaining measurement data $y_{t}^{\left(\mathrm{mis}\right)}\in R$ are conceptual and denote the data that were not observed.

For the initial state,

(8) $$p\left(x_{1}\right)=N\left(x_{1};\mu_{1},\Sigma_{1}\right).$$

**Figure 1:**
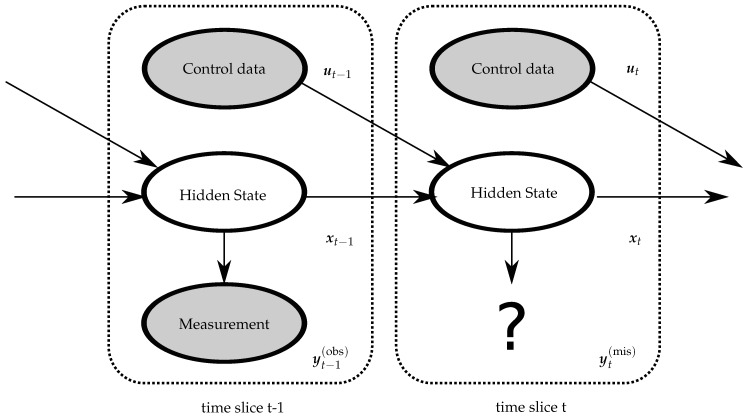
Two time slice Bayesian network with missing measurement data.

With the state transition model defined in (6) and the emission model in (7), we are now ready to derive the DBN as illustrated by two time slices in Figure 1.

In the sequel, the parameter vector $\theta =\{A,B,,,\Sigma_{w},\mu_{1},\Sigma_{1}\}$, the latent state sequence $x≜\{x_{1},\ldots ,x_{T}\}$, and the missing data are unknown and, hence, require estimation.

#### 2.2.1. The EM Algorithm

This section derives a maximum-likelihood based tracking algorithm for the state sequence. So far, we have designed the DBN in Figure 1. Unrolling the DBN for *T* time slices, it follows for the joint state-measurement probability distribution that:

(9) $$p\left(x,y^{\left(\mathrm{obs}\right)},y^{\left(\mathrm{mis}\right)}|\theta \right)=p\left(x_{1}|\theta \right)p\left(y_{1}^{\left(\mathrm{obs}\right)},y_{1}^{\left(\mathrm{mis}\right)}|x_{1},\theta \right)\prod_{t=2}^{T}p\left(x_{t}|x_{t-1},\theta \right)p\left(y_{t}^{\left(\mathrm{obs}\right)},y_{t}^{\left(\mathrm{mis}\right)}|x_{t},\theta \right).$$

Due to the Markov property in (6), the joint state-measurement distribution decomposes into the product of individual conditional distributions. We now have two options for handling missing observations: (i) modeling the missing data mechanism or (ii) ignoring it, which is equivalent to integrating out the missing observations from the joint density function in (9). Following this approach, we get:

(10) $$p\left(x,y^{\left(\mathrm{obs}\right)}|\theta \right)=\int p\left(x,y^{\left(\mathrm{obs}\right)},y^{\left(\mathrm{mis}\right)}|\theta \right)dy^{\left(\mathrm{mis}\right)}.$$

Rubin showed in [20] that sufficient conditions for ignoring missing data are:

- The data are MAR, i.e., the missing data mechanism is only allowed to depend on $y^{\left(\mathrm{obs}\right)}$;
- the model parameters governing absence and the parameters of interest θ reside in different spaces.

In our case, both conditions are fulfilled, so that we may safely ignore the missing data mechanism.

Based on the marginal distribution in (10), we now use the EM algorithm to approximate the Bayesian inference of the state sequence. The EM algorithm postulates complete (hidden) data X that would ease the computation of θ if they were known. Following this approach, let X contain the reciprocal dynamics of plant growth along with the observed measurement data, i.e., $X=\{x,y^{\left(\mathrm{obs}\right)}\}$. Starting from iteration i=0, the E-step of the algorithm provides the expected value of the entire complete data given the observed measurements and a guess of the parameter vector, i.e.,

(11) $$Q\left(\theta |\theta^{\left[i\right]}\right)=E\left\{\ln p\left(x,y^{\left(\mathrm{obs}\right)}|\theta \right)|y^{\left(\mathrm{obs}\right)},\theta^{\left[i\right]}\right\}.$$

The E-step iterates between prediction and correction if measurement data are available or only predicts the complete data based on previous state information without a response when measurement data are missing according to (10). The M-step,

(12) $$\theta^{\left[i+1\right]}=\arg\max_{\theta }Q\left(\theta |\theta^{\left[i\right]}\right),$$

is learning from the updated complete data in the E-step. The sequence of log-likelihood values ${\left\{\ln p\left(y^{\left(\mathrm{obs}\right)}|\theta^{\left[i\right]}\right)\right\}}_{i=0}^{\infty }$ is non-decreasing and converges to a stationary point [24,41].

At iterative convergence, the expected state sequence has the form:

(13) $$x_{t}^{\left[\infty \right]}=E\left\{x_{t}|y^{\left(\mathrm{obs}\right)},\theta^{\left[\infty \right]}\right\}$$

with the error variance:

(14) $$V_{t}^{\left[\infty \right]}=\mathrm{Cov}\left\{x_{t}|y^{\left(\mathrm{obs}\right)},\theta^{\left[\infty \right]}\right\}.$$

The derivation of (13) and (14) is provided in Appendix A.

#### 2.2.2. Prediction of Measurement Data

If control data were available *q* days beyond the current growing day *T*, but measurement is missing, the expected state-sequence in (13)) would already be the solution to our problem. In practice, however, control data are only available until time step *T*. A simple yet efficient predictor is to deploy (13), but keep control data frozen at time step *T*. Following this approach, we obtain:

(15) $$x_{T+q}^{\left[\infty \right]}=\left(I-A^{\left[\infty \right]}U_{T}\right)x_{T+q-1}^{\left[\infty \right]}+B^{\left[\infty \right]}u_{T}.$$

Substituting (15) for (7), it follows for the *q*-step ahead predictor of the measurement data that:

(16) $$y_{T+q}^{\left[\infty \right]}=C^{\left[\infty \right]}\left(I-A^{\left[\infty \right]}U_{T}\right)x_{T+q-1}^{\left[\infty \right]}+C^{\left[\infty \right]}B^{\left[\infty \right]}u_{T}.$$

having error variance:

(17) $$\Sigma_{y,T+q}^{\left[\infty \right]}=C^{\left[\infty \right]}\left(\left(I-A^{\left[\infty \right]}U_{T}\right)V_{T-1+q}{\left(I-A^{\left[\infty \right]}U_{T}\right)}^{T}+\Sigma_{n}^{\left[\infty \right]}\right){\left(C^{\left[\infty \right]}\right)}^{T}.$$

The above predictor runs freely without response.

#### 2.2.3. Initialization of the EM Algorithm

The stationary point of the log-likelihood function, reached by the EM algorithm, highly depends on the initial point [42]. Hence, initialization requires special care.

For the noise covariances, we start off with small values on their diagonal:

(18) $$\Sigma_{n}^{\left[0\right]}=\Sigma_{w}^{\left[0\right]}=\Sigma_{1}^{\left[0\right]}=\epsilon I,\;\epsilon \ll 1;$$

The measurement matrix $C^{\left[0\right]}$ is initialized as:

(19) $$C^{\left[0\right]}=E$$

where E is the all-one matrix. Substituting $C^{\left[0\right]}$ for (8), the state-sequence starts off with:

(20) $$\mu_{1}^{\left[0\right]}={\left(C^{\left[0\right]}\right)}^{T}{\left(C^{\left[0\right]}{\left(C^{\left[0\right]}\right)}^{T}\right)}^{-1}y_{1}^{\left(\mathrm{obs}\right)}.$$

The initial matrices $A^{\left[0\right]}$ and $B^{\left[0\right]}$ depend on the growth parameter. For the LAI, the reciprocal logistic curve in (3) is a function of three parameters κ, μ, and $t_{0}$, which are the solutions to three equations:

(21) $$\begin{array}{c} \begin{array}{cc} \mu & =\frac{\log\left(y_{t_{2}}^{\left(\mathrm{obs}\right)}/\kappa -1\right)-\log\left(y_{t_{1}}^{\left(\mathrm{obs}\right)}/\kappa -1\right)}{t_{1}-t_{2}}\\ t_{0} & =\frac{\log\left(y_{t_{1}}^{\left(\mathrm{obs}\right)}/\kappa -1\right)+\mu t_{1}}{\mu }\\ \kappa & =\frac{y_{t_{3}}^{\left(\mathrm{obs}\right)}}{1+\exp\left\{-\mu (t_{3}-t_{0})\right\}} \end{array} \end{array}$$

This system of nonlinear equations, however, has no closed-form solution. For MicroTom tomatoes, the reciprocal LAI has the steady state of κ≈1 [43]. Having fixed κ, the computation of $t_{0}$ is obsolete, and μ has the closed-form solution in (21). From (4) and (6), it can be seen that the state matrix A is proportional to the decay. Ergo,

(22) $$A^{\left[0\right]}=\mu \;\mathrm{diag}\{\frac{1}{T}\sum_{t=1}^{T}u_{t}{\}}^{-1}.$$

We normalized $A^{\left[0\right]}$ by the sample mean of the control signal. As the control matrix B is proportional to the decay and the steady state, it follows:

(23) $$B^{\left[0\right]}=A^{\left[0\right]}\kappa .$$

For the ET, the negative exponential decay in (5) can be specified by two observation points according to:

(24) $$\begin{array}{c} \begin{array}{cc} \mu^{\prime } & =-\frac{1}{t_{1}}\left(\log(1-y_{t_{1}}^{\left(\mathrm{obs}\right)}/\kappa^{\prime })\right)\\ \kappa^{\prime } & =\frac{y_{t_{2}}^{\left(\mathrm{obs}\right)}}{1-\exp\left\{-\mu^{\prime }t_{2}\right\}} \end{array} \end{array}$$

which can be solved in an iterative fashion. Substituting $\mu^{\prime }$ for μ in (22), as well as $\kappa^{\prime }$ for κ in (23), we again obtain initial guesses for the matrices $A^{\left[0\right]}$ and $B^{\left[0\right]}$, respectively.

With this type of initialization, our EM algorithm is able to find the global maximum of the likelihood function with the aid of only two initial observations.

### 2.3. Experimental Design

Trials were conducted at the Department of Agriculture, Food and Environment at the University of Pisa, Pisa, Italy (latitude 43°42′ N, longitude 10°24′ E). MicroTom plants were cultivated in plastic planter pots 10 × 10 × 17 cm filled with perlite/peat moss at a density of 12 plants m${}^{-2}$ The plants were irrigated with a nutrient solution with the following nutrient ion composition (expressed as mol m${}^{-3}$): N-NO${}_{3}^{-}$ 10.4, H${}_{2}$PO${}_{4}^{-}$ 1.0, K${}^{+}$ 7.5, Mg${}^{2+}$ 2.0, Ca${}^{2+}$ 4.5, plus Hoagland concentration of trace elements. The Electrical Conductivity (EC) of the nutrient solution was 2.34 dS m${}^{-1}$, and the pH was 5.5. The experiments were conducted between 1 April 2019 and 31 May 2019 in three different experimental environments. Environment 1 (Env 1) was a warm greenhouse with a high level of light; Environment 2 (Env 2) was shaded and equipped with emergency heating only; while Environment 3 (Env 3) was a first prototype of a domestic greenhouse for indoor cultivation equipped with LED lighting system. The three growth environments were chosen such that the growth of MicroTom could be evaluated at different conditions of light and temperature, which are the main parameters influencing crop development and crop evapotranspiration. The environmental parameters temperature converted into Growing Degree Days (GDD) and the solar irradiance *R* were sensed (Netsens, Sesto Fiorentino, Tuscany, Italy) and recorded (Netsens Wireless Unit Model No. MN-0086-AE). Table 1 lists their descriptive statistics.

The different climatic conditions produced important differences in cumulated ET and growth. The ET was measured in the mornings by using a scale. Roughly 50 percent of all data points, however, were missing. Leaf area measurements were made on 5 plants as the sum of the area of the leaves of each plant by a planimeter (DT Area Meter MK2, Delta T-Devices, Cambridge, U.K.), starting from Growing Days after Transplantation (GDT) equal to zero and ending at the ultimate GDT with two samples in between.

The proposed DBN was benchmarked against the non-linear growth model by Carmassi in Section 2.1, as well as a low-complex Linear time-series Regression Model (LRM). For round-fruit tomatoes (*Solanum lycopersicum* L. cv. Jama F1), the parameters of the model were given in [28]. Since the vegetative habitus of our cv. “MicroTom” was quite different, it was necessary to re-calibrate the model parameters. Using the dataset of Env 1, it followed for the LAI reference model in (1) by Carmassi that α=0.03, β=0.90, ζ=560, and δ=50 when the base temperature was 10 °C. Following the same approach for the ET reference model in (2), we obtained ϵ=0.109 and γ=0.32 mm d${}^{-1}$.

The competing LRM used the same training data as our DBN did. Due to lack of measurement data, the LRM describes a linear relationship between LAI and cumulative GDD, only, according to:

(25) $${\mathrm{LAI}}_{t}=\rho_{0}+\rho_{1}\sum_{t^{\prime }\leq t}{\mathrm{GDD}}_{t^{\prime }}+\varepsilon_{t}$$

where $\rho_{0}$ and $\rho_{1}$ are the regression coefficients using a standard least squares fit and ε is the model error. For the ET, the LRM assumes a linear relation between the dependent variable ET and the independent variables GDD accumulated over time and *R* according to:

(26) $${\mathrm{ET}}_{t}=\rho_{0}+\rho_{1}\sum_{t^{\prime }\leq t}{\mathrm{GDD}}_{t^{\prime }}+\rho_{2}R_{t}+\varepsilon_{t}.$$

Finally, it should be noted that the EM algorithm in our DBN was iterated until the log-likelihood function $\ln p\left(y^{\left(\mathrm{obs}\right)}|\theta^{\left[i\right]}\right)$ increased by less than $1{}^{o}\;/{\;}_{\mathrm{oo}}$ or the limit of 100 iterations was reached.

## 3. Results and Discussion

Given the environmental parameters in Section 2.3 and sparse historical measurement data until some time instant *T*, our DBN was deployed to predict the posterior distribution *q* growing days ahead.

**Figure 2:**
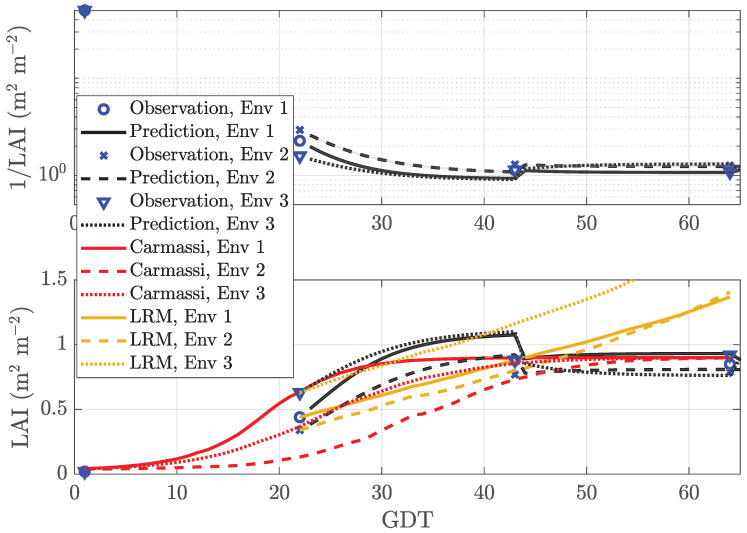
Prediction of the LAI with prediction length q=21 for micro-tomatoes (MicroTom) with sparse data in three environments: Env 1 (warm and bright), Env 2 (shaded and emergency heating), and Env 3 (indoor and artificial illumination). LRM, Linear time-series Regression Model.

In MicroTom LAI prediction, the measurement data were observed in only 6% of all cases. The other 94 % were treated as MAR. The control data, however, were available at all time instants. The EM algorithm operated on the reciprocal LAI. The first two measurement points were used to initialize the algorithm according to Section 2.2.3 with κ=1. Figure 2 reports the performance of our DBN as a function of GDT. At the day of measurement, our predictor forecast the next measurement, which was q=21 days (very long) ahead. In the upper subplot, the blue markers indicate the reciprocal observations at T={1,22,43,64}. The black curves show the mean prediction value $y_{T+21}^{\left[\infty \right]}$ of 1/LAI in (16). Its standard deviation $\Sigma_{T+21}^{\left[\infty \right]}$ in (17) was encoded as half the width of the filled region around the mean value. Clearly, the predictor only had access to historical measurement data until time instant *T*. The lower subplot shows the LAI vs. GDT using the same line-styles. For comparison purposes, the Carmassi model in (1) and the LRM in (25) are also added to the plot as red and brown lines, respectively. It can be seen that our DBN correctly anticipated the reflection point of the sigmoid function in all three cultivation environments despite the extremely low number of measurement points. Clearly, with the increasing number of observed data points, the quality of the LAI forecasts improved. The average prediction error, averaged over all *T*, was equal to 15.5%, 12.2%, and 19.7% for Env 1, Env 2, and Env 3, respectively. Details are listed in Table 2. Note that the inverse of the reciprocal mean depicted in the lower subplot is a lower bound of the mean value shown in the upper subplot (Jensen’s inequality). Moreover, the error variance of the reciprocal of a Gaussian random variable tends to infinity and, hence, was omitted from the lower subplot. The analytic Carmassi model accurately estimated the inflection point in Env 3 excluding the reference environment Env 1. The simple LRM in (25) could be seen as first-order approximation of the former in (1) and, hence, was quite accurate in the neighborhood of the inflection point of the sigmoid function, but became more erroneous the larger the time deviation was.

**Figure 3:**
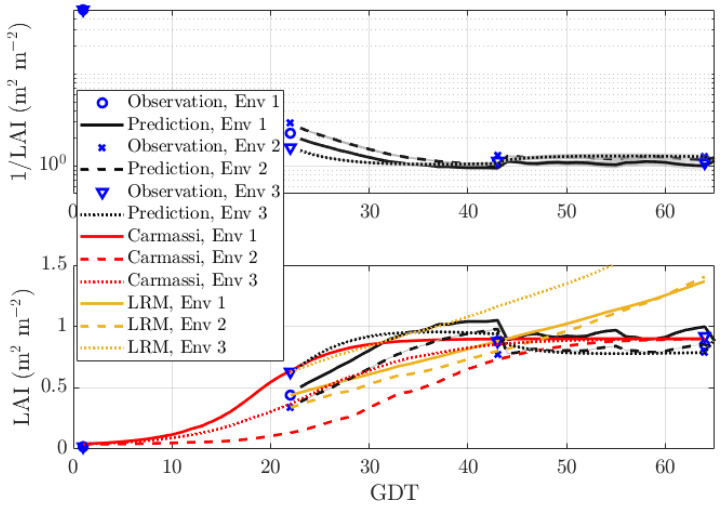
Prediction of the LAI with prediction length q=1 for MicroTom with sparse data in three environments: Env 1 (warm and bright), Env 2 (shaded and emergency heating), and Env 3 (indoor and artificial illumination).

Figure 3 shows the performance of our DBN with prediction length q=1 day (very short). At time *T*, the EM algorithm either observed or missed the measurement point, requiring reconstruction. The resulting curve, influenced by the different control data every day, was rougher, but generally speaking, more accurate than for a prediction length of q=21. In particular for Env 3, the prediction error was now under 10%.

Table 3 indicates that the mean estimation error for the analytical Carmassi model was equal to 19.42% (13.46%) for Env 2 (Env 3). This error was dominated by the loose approximation near the initial growth state. The mean estimation error of the LRM was equal to 23.88 %, 34.0%, and 50.0% for Env 1, Env 2, and Env 3, respectively. In contrast to the Carmassi model, this error was dominated by the steady growth state farthest away from the likely missed inclination point due to the nature of the first order approximation.

**Figure 4:**
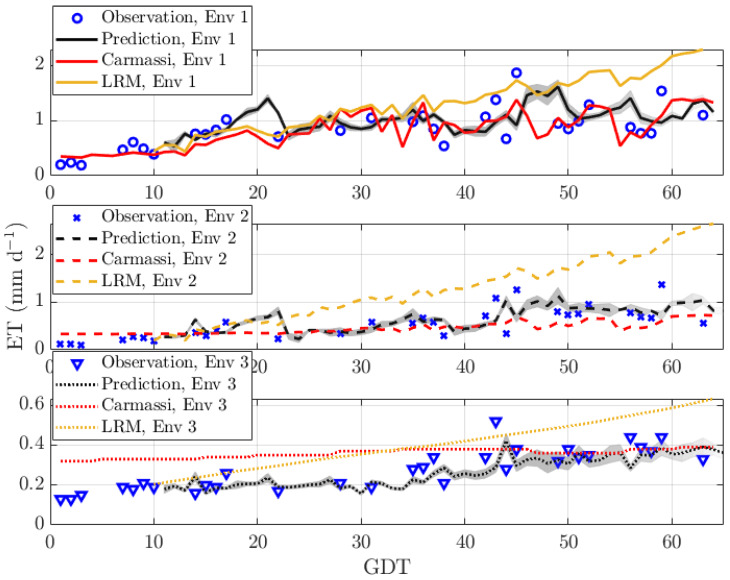
Prediction of the ET for MicroTom with sparse data in three environments: Env 1 (warm and bright), Env 2 (shaded and emergency heating), and Env 3 (indoor and artificial illumination).

Figure 4 reports the ET vs. GDT for our MicroTom in all three environments. The upper, middle, and lower sub-plots represent the greenhouse environments Env 1, Env 2, and Env 3, respectively. This time, the EM algorithm operated directly on the ET (and not on its reciprocal). In this scenario, measurement data were missing roughly 50% of the time. The start of the prediction was at T=10. The prediction length q=1. It can be seen that our predictor was able to follow the trend of the measurement curve. It predicted small outliers caused by changes in the environmental parameters with increasing accuracy the longer the observation time was. However, it had difficulties with anticipating large outliers. This was true for all environments. The Carmassi model in (2) was only quite accurate for Env 1, on which it was calibrated. The linear dependency of the LRM in (26) overemphasized the impact of the environmental parameters on the ET, in particular for Env 2.

**Figure 5:**
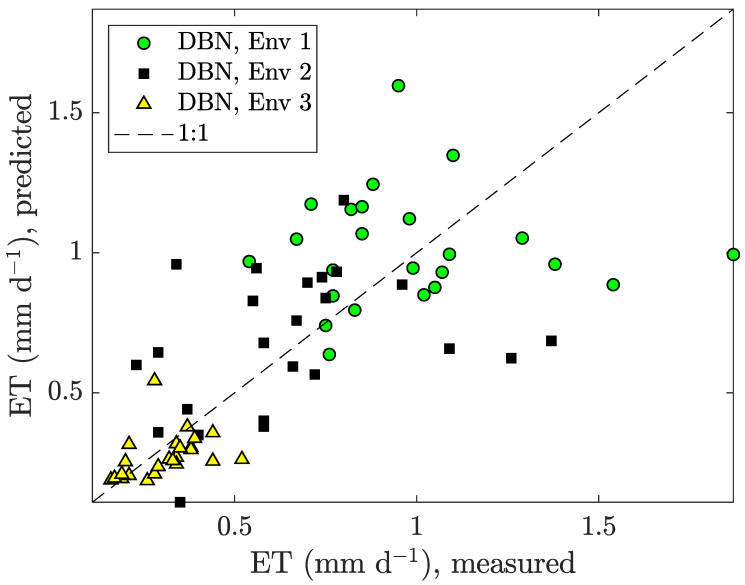
Measured vs. predicted measurement data obtained by our DBN application for the micro-tomatoes in three different environments: Env 1 (warm and bright), Env 2 (shaded and emergency heating), and Env 3 (indoor and artificial illumination).

Figure 5 illustrates the measured cumulative ET vs. the predicted for our predictor in (16). It can be seen that the predicted values were roughly Gaussian distributed around the 1:1 line, indicating freedom from bias. This was true for all three environments. The average prediction error of our DBN application was 29.42%.

Not shown in the already overcrowded plot, the Carmassi model and the LRM had average prediction errors of 37.21% and 67.23%, respectively. Thus, it could be concluded that our DBN performs similar to the analytical Carmassi model. Error performance details are listed in Table 4.

## 4. Conclusions

We proposed a Bayesian machine learning approach for the inference of dynamical systems that modeled crop growth indicators with a sigmoid evolution over time. Such an evolution essentially corresponded to a two-phase dynamical process consisting of an exponential growth of the plant followed by a slow-down, leading to the achievement of a steady state. Both LAI and ET, which are among the most common growth-related indicators, exhibit such a dynamics for some types of plants.

The main advantage of our approach was flexibility. The model was inferred from the first items of the time series of indicators and environmental parameters, and it could be used to predict future items. This enabled real-time estimation of crop growth without the need to dedicate a whole cultivation cycle to a species-specific model calibration.

Compared to analytical models based on regression, which can fit calibration data in a very accurate way, our approach turned out to be more robust to changes in the environmental parameters. Again, this was a consequence of the fact that the model learned the influence of environmental parameters on the growth indicators from (the first items of) the same time series it would have to predict.

The proposed approach could also take advantage of available information about the usual trend of the growth indicators of interest. For instance, in the considered MicroTom tomatoes example, we used knowledge on the reciprocal LAI steady state (usually close to one) to initialize the matrices for the learning phase properly. This allowed us to obtain a more accurate inference of the dynamical model, and consequently more accurate predictions. Generalizing, this showed that the approach could also take available agronomic knowledge into account in order to improve the accuracy of predictions. For example, another piece of information that could be used, if available, was the usual time the steady state was expected to be reached (i.e., the expected length of the cultivation cycle). As future work, we plan to investigate how we could transform this information into an additional constraint for the learning phase and to measure the effect of such a constraint on the prediction capability of the inferred model.

## Figures and Tables

**Table 1:** Descriptive statistics of the control parameters in three different climatic environments. Env, Environment.

		Temperature	GDD	Irradiance *R*
		(°C)	(C°)	(MJ m${}^{-2}$ d${}^{-1}$)
Env 1	Mean	22.86	12.86	12.82
	Maximum	27.29	17.3	21.18
	Minimum	18.59	8.6	2.35
	Accumulation	N/A	1158.7	820.67
Env 2	Mean	18.40	8.4	4.70
	Maximum	24.57	14.6	8.20
	Minimum	12.08	2.1	1.49
	Accumulation	N/A	857.2	291.44
Env 3	Mean	19.14	9.14	1.29
	Maximum	23.63	13.60	1.29
	Minimum	15.31	5.30	1.29
	Accumulation	N/A	921.1	82.56

**Table 2:** Prediction error of LAI vs. GDT for MicroTom with sparse data in three environments: Env 1 (warm and bright), Env 2 (shaded and emergency heating), and Env 3 (indoor and artificial illumination).

Error (%)	q=1		q=21	
	T=43	T=64	T=43	T=64
DBN Env 1	16.86	13.03	22.48	9.17
DBN Env 2	25.64	6.36	21.30	2.74
DBN Env 3	8.34	9.68	23.74	14.82

**Table 3:** Prediction error of the Carmassi and the Linear Regression Model (LRM) for LAI vs. GDT. The parameters of the former were adjusted during the cultivation cycle in Env 1, while the slope and intercept of the latter during the first two measurement points in each cultivation cycle.

Error (%)	T=1	T=22	T=43	T=64
Carmassi Env 2	102.76	55.61	16.44	13.92
Carmassi Env 3	109.19	41.55	2.90	2.24
LRM Env 1	N/A	N/A	0	61.0
LRM Env 2	N/A	N/A	4.05	77.82
LRM Env 3	N/A	N/A	32.19	101.26

**Table 4:** Estimation error of ET vs. GDT for the micro-tomatoes in three different environments: Env 1 (warm and bright), Env 2 (shaded and emergency heating), and Env 3 (indoor and artificial illumination).

Error (%)	Env 1	Env 2	Env 3
DBN	30.13	37.54	20.57
Carmassi	N/A	37.85	36.57
LRM	49.56	112.21	39.92

## Abbreviations

The following abbreviations are used in this manuscript:

AI	Artificial Intelligence
COLTIV@MI	COLTIVazione Automatizzata Miniaturizzata Innovativa
DBN	Dynamic Bayesian Network
EM	Expectation-Maximization
ET	Evapotranspiration
GDD	Growing Degree Days
GDT	Growing Days after Transplantation
ICT	Information and Communication Technology
IoT	Internet of Things
LAI	Leaf Area Index
MAR	Missing At Random
MCAR	Missing Completely At Random
RBF	Radial Basis Function
SVR	Support Vector Regression

## Appendix A. Application of the EM Algorithm to Plant Growth Tracking

In this Appendix, we derive the EM algorithm for the state-space model in (6) and (7) when measurement data are missing at random.

From (10), the log-likelihood function of θ for the complete data X is given by:

(A1) $$\begin{array}{c} \begin{array}{cc} \ln p\left(X|\theta \right)\propto & -\ln|\Sigma_{1}|-{\left(x_{1}-\mu_{1}\right)}^{T}\Sigma_{1}^{-1}\left(x_{1}-\mu_{1}\right)\\ \; & -T\ln|\Sigma_{w}|-\sum_{t=1}^{T}{\left(y^{\left(\mathrm{obs}\right)}t-Cx_{t}\right)}^{T}\Sigma_{w}^{-1}\left(y^{\left(\mathrm{obs}\right)}t-Cx_{t}\right)\\ & -(T-1)\ln|\Sigma_{n}|\\ & -\sum_{t=2}^{T}{\left(x_{t}-\left(I-A\;U_{t-1}\right)x_{t-1}-Bu_{t-1}\right)}^{T}\Sigma_{n}^{-1}\left(x_{t}-\left(I-A\;U_{t-1}\right)x_{t-1}-Bu_{t-1}\right). \end{array} \end{array}$$

Substituting (A1) for the expected log-likelihood function function in (11), the result depends on the four conditional expectations:

(A2) $$\begin{array}{c} \begin{array}{cc} x_{t}^{\left[i\right]} & ≜E\{x_{t}|y^{\left(\mathrm{obs}\right)},\theta^{\left[i\right]}\}\\ V_{t}^{\left[i\right]} & ≜\mathrm{Cov}\{x_{t}|y^{\left(\mathrm{obs}\right)},\theta^{\left[i\right]}\}\\ {\left(x_{t}x_{t}^{T}\right)}^{\left[i\right]} & ≜E\left\{x_{t}x_{t}^{T}|y^{\left(\mathrm{obs}\right)},\theta^{\left[i\right]}\right\}=V_{t}^{\left[i\right]}+x_{t}^{\left[i\right]}{\left(x_{t}^{\left[i\right]}\right)}^{T}\\ {\left(x_{t}x_{t-1}^{T}\right)}^{\left[i\right]} & ≜E\left\{x_{t}x_{t-1}^{T}|y^{\left(\mathrm{obs}\right)},\theta^{\left[i\right]}\right\}=V_{t}^{\left[i\right]}J_{t-1}^{T}+x_{t}^{\left[i\right]}{\left(x_{t-1}^{\left[i\right]}\right)}^{T}, \end{array} \end{array}$$

with the short-cut:

(A3) $$J_{t-1}≜\mathrm{Cov}\left\{x_{t-1},x_{t}|y^{\left(\mathrm{obs}\right)},\theta^{\left[i\right]}\right\}\mathrm{Cov}{\left\{x_{t}|y^{\left(\mathrm{obs}\right)},\theta^{\left[i\right]}\right\}}^{-1}.$$

The forward-backward algorithm [44,45] can be used to efficiently compute the expectations in (A2). Following the approach in [19], the forward recursion of the forward-backward algorithm yields:

(A4) $$\begin{array}{c} \begin{array}{cc} \mu_{t} & ≜m_{t-1}+\left\{\begin{array}{ccc} K_{t}\left(y_{t}-C^{\left[i\right]}m_{t-1}\right) & ; & y_{t}\in y^{\left(\mathrm{obs}\right)}\\ 0 & ; & y_{t}\in y^{\left(\mathrm{mis}\right)} \end{array}\right.\\ V_{t} & ≜\left(I-K_{t}C^{\left[i\right]}\right)\left(\left(I-A^{\left[i\right]}\;U_{t-1}\right)V_{t-1}{\left(I-A^{\left[i\right]}\;U_{t-1}\right)}^{T}+\Sigma_{n}^{\left[i\right]}\right). \end{array} \end{array}$$

with the Kalman gain matrix K:

(A5) $$K_{t}≜P_{t-1}{\left(C^{\left[i\right]}\right)}^{T}{\left[C^{\left[i\right]}P_{t-1}{\left(C^{\left[i\right]}\right)}^{T}+\Sigma_{w}^{\left[i\right]}\right]}^{-1},$$

and the short-cuts:

(A6) $$m_{t}≜\left(I-A^{\left[i\right]}\;U_{t}\right)\mu_{t}+B^{\left[i\right]}u_{t}$$

and:

(A7) $$P_{t}≜\left(I-A^{\left[i\right]}\;U_{t}\right)V_{t}{\left(I-A^{\left[i\right]}\;U_{t}\right)}^{T}+\Sigma_{n}^{\left[i\right]}.$$

The backward recursion, in contrast, yields the state estimate of interest, namely a normal distribution with mean and covariance:

(A8) $$\begin{array}{c} \begin{array}{cc} x_{t}^{\left[i\right]}= & \mu_{t}+J_{t}\left(x_{t+1}^{\left[i\right]}-m_{t}\right),\\ V_{t}^{\left[i\right]}= & V_{t}+J_{t}\left(V_{t+1}^{\left[i\right]}-P_{t}\right)J_{t}^{T}, \end{array} \end{array}$$

respectively, where:

(A9) $$J_{t}=V_{t}{\left(I-A^{\left[i\right]}\;U_{t}\right)}^{T}P_{t}^{-1}.$$

Finally, the boundary conditions are updated according to:

(A10) $$\begin{array}{cc} x_{T}^{\left[i\right]} & =\mu_{T}\\ V_{T}^{\left[i\right]} & =V_{T}. \end{array}$$

The M-step of the EM algorithm in (11) learns the parameter vector θ by computing the partial derivative of the expected log-likelihood function in (11), setting the result equal to zero, and solving with respect to the respective parameter, leaving:

(A11) $$\begin{array}{c} \begin{array}{cc} B^{\left[i+1\right]}= & \left[\sum_{t=2}^{T}\left(x_{t}^{\left[i\right]}-x_{t-1}^{\left[i\right]}\right)u_{t-1}^{T}-\left({\left(x_{t}x_{t-1}^{T}\right)}^{\left[i\right]}-{\left(x_{t-1}x_{t-1}^{T}\right)}^{\left[i\right]}\right)U_{t-1}\right]{\left[\sum_{t=2}^{T}U_{t-1}{\left(x_{t-1}x_{t-1}^{T}\right)}^{\left[i\right]}U_{t-1}\right]}^{-1}\\ \; & \times \left[\sum_{t=2}^{T}U_{t-1}x_{t-1}^{\left[i\right]}u_{t-1}^{T}\right]\\ \; & \times {\left[\sum_{t=2}^{T}u_{t-1}u_{t-1}^{T}-\sum_{t=2}^{T}u_{t-1}{\left(x_{t-1}^{T}\right)}^{\left[i\right]}U_{t-1}{\left(\sum_{t=2}^{T}U_{t-1}{\left(x_{t-1}x_{t-1}^{T}\right)}^{\left[i\right]}U_{t-1}\right)}^{-1}\sum_{t=2}^{T}U_{t-1}x_{t-1}^{\left[i\right]}u_{t-1}^{T}\right]}^{-1} \end{array} \end{array}$$

(A12) $$\begin{array}{c} \begin{array}{cc} A^{\left[i+1\right]}= & \left[\sum_{t=2}^{T}\left({\left(x_{t-1}x_{t-1}^{T}\right)}^{\left[i\right]}-{\left(x_{t}x_{t-1}^{T}\right)}^{\left[i\right]}\right)U_{t-1}+B^{\left[i+1\right]}u_{t-1}{\left(x_{t-1}^{T}\right)}^{\left[i\right]}U_{t-1}\right]\\ \; & \times {\left[\sum_{t=2}^{T}U_{t-1}{\left(x_{t-1}x_{t-1}^{T}\right)}^{\left[i\right]}U_{t-1}\right]}^{-1} \end{array} \end{array}$$

(A13) $$\begin{array}{c} \begin{array}{cc} \Sigma_{n}^{\left[i+1\right]}= & \frac{1}{T-1}\sum_{t=2}^{T}{\left(x_{t}x_{t}^{T}\right)}^{\left[i\right]}-\left(I-A^{\left[i+1\right]}U_{t-1}\right){\left(x_{t-1}x_{t}^{T}\right)}^{\left[i\right]}-B^{\left[i+1\right]}u_{t-1}{\left(x_{t}^{T}\right)}^{\left[i\right]}\\ \; & +\left[{\left(x_{t}x_{t-1}^{T}\right)}^{\left[i\right]}-\left(I-A^{\left[i+1\right]}U_{t-1}\right){\left(x_{t-1}x_{t-1}^{T}\right)}^{\left[i\right]}-B^{\left[i+1\right]}u_{t-1}{\left(x_{t-1}^{T}\right)}^{\left[i\right]}\right]\left(I-U_{t-1}{\left(A^{T}\right)}^{\left[i+1\right]}\right)\\ \; & \left(-x_{t}^{\left[i\right]}+\left(I-A^{i+1]}U_{t-1}\right)x_{t-1}^{\left[i\right]}+B^{\left[i+1\right]}u_{t-1}\right)u_{t-1}^{T}{\left(B^{T}\right)}^{\left[i+1\right]} \end{array} \end{array}$$

(A14) $$\begin{array}{c} \begin{array}{cc} C^{\left[i+1\right]}= & \left(\sum_{t\in \mathrm{obs}}y_{t}^{\left(\mathrm{obs}\right)}{\left(x_{t}^{\left[i\right]}\right)}^{T}\right){\left(\sum_{t\in \mathrm{obs}}^{T}{\left(x_{t}x_{t}^{T}\right)}^{\left[i\right]}\right)}^{-1}. \end{array} \end{array}$$

(A15) $$\begin{array}{c} \begin{array}{cc} \Sigma_{w}^{\left[i+1\right]}= & \frac{1}{T^{\left(\mathrm{obs}\right)}}\sum_{t=1}^{T}y_{t}^{\left(\mathrm{obs}\right)}{\left(y_{t}^{\left(\mathrm{obs}\right)}\right)}^{T}-y_{t}^{\left(\mathrm{obs}\right)}{\left(x_{t}^{T}\right)}^{\left[i\right]}{\left(C^{T}\right)}^{\left[i+1\right]}-C^{\left[i+1\right]}\left(x_{t}^{\left[i\right]}{y_{t}^{\left(\mathrm{obs}\right)}}^{T}-{\left(x_{t}x_{t}^{T}\right)}^{\left[i\right]}\right){\left(C^{\left[i+1\right]}\right)}^{T}, \end{array} \end{array}$$

(A16) $$\begin{array}{c} \begin{array}{cc} \mu_{1}^{\left[i+1\right]}= & x_{1}^{\left[i\right]}, \end{array} \end{array}$$

(A17) $$\begin{array}{c} \begin{array}{cc} \Sigma_{1}^{\left[i+1\right]}= & {\left(x_{1}x_{1}^{T}\right)}^{\left[i\right]}-\mu_{1}^{\left[i+1\right]}{\left(\mu_{1}^{\left[i+1\right]}\right)}^{T}=V_{1}^{\left[i\right]}. \end{array} \end{array}$$

Note that all the parameters related to the measurements in (A13) and (A14) are based on the observed measurement data, ignoring the missing data. The above state and parameter estimates are the base of our DBN in Section 2.2.
